# The Role of Hyponatremia in Identifying Complicated Cases of Acute Appendicitis in the Pediatric Population

**DOI:** 10.3390/diagnostics15111384

**Published:** 2025-05-30

**Authors:** George Kottakis, Konstantina Bekiaridou, Stylianos Roupakias, Orestis Pavlides, Ioannis Gogoulis, Spyridon Kosteletos, Theodoros Nektarios Dionysis, Aggelos Marantos, Katerina Kambouri

**Affiliations:** 1 Pediatric Surgery Department, Elena Venizelou General Hospital, 115 21 Athens, Greece; 2 Pediatric Surgery Department, University Hospital of Alexandroupolis, Democritus University of Thrace, 681 00 Alexandroupolis, Greece; bekiaridounadia@hotmail.com (K.B.); stylroup@yahoo.gr (S.R.); ioannis_gogoulis@hotmail.com (I.G.); 3 1st Pediatric Surgery Department, Panagiotis and Aglaia Kyriakou Children’s Hospital of Athens, 115 27 Athens, Greece; orepavlides@gmail.com (O.P.); kosteletosspyros@gmail.com (S.K.); thdionysis@gmail.com (T.N.D.); aggelosmarados@gmail.com (A.M.)

**Keywords:** hyponatremia, acute complicated appendicitis, pediatric surgery

## Abstract

**Background**: Hyponatremia has been identified as a marker of disease severity in various inflammatory conditions. However, its role in predicting acute complicated appendicitis (ACA) in children remains under investigation. This study evaluated the association between preoperative hyponatremia and ACA in a pediatric population. **Methods**: A retrospective study was conducted on pediatric patients treated for acute appendicitis in two major pediatric centers in Greece. Patients were categorized into groups based on the presence of acute uncomplicated appendicitis (AUA) and acute complicated appendicitis (ACA). Preoperative laboratory parameters were analyzed to identify potential predictors of ACA. **Results**: This study included 491 pediatric patients, with a mean age of 10 years. ACA patients exhibited significantly lower Na levels compared to those with AUA (136 vs. 138 mmol/L, *p* < 0.001). Hyponatremia (<135 mmol/L) was present in 38.4% of ACA cases compared to 2.2% of AUA cases (*p* < 0.001), and was associated with a significantly increased risk of ACA (OR = 18.30, *p* < 0.001). A sodium threshold of 135 mmol/L also demonstrated a sensitivity of 48% and a specificity of 92.1% **Conclusions**: Hyponatremia is a strong and specific predictor of ACA in children. When combined with other inflammatory markers, it may enhance early risk stratification, aiding in timely surgical decision making.

## 1. Introduction

Acute appendicitis (AA) remains one of the most common surgical emergencies encountered in the pediatric population. The annual rate of AA in the United States is 9.38 cases per 10,000, with a peak incidence observed among 10–19-year-olds [1]. Traditionally, it has been assumed that all cases of appendicitis will ultimately lead to rupture if left untreated, necessitating surgical intervention as the definitive treatment. However, recent studies challenge this assumption, indicating that patients with uncomplicated appendicitis may be managed conservatively without the significant risk of complications, marking a major change in course in the therapeutic algorithm and allowing these patients to avoid the inherent risks of surgical management [2].

AA is initiated by the obstruction of the appendiceal lumen, leading to increased intraluminal pressure, vascular compromise, bacterial overgrowth and eventual inflammation [3]. If left unchecked, this cascade can progress to gangrene, perforation and generalized peritonitis [3]. Surgical intervention, when needed, is typically performed either through open or laparoscopic appendectomy. While laparoscopic surgery offers advantages such as reduced postoperative pain and quicker recovery [4], open appendectomy is still widely employed, especially in institutions where laparoscopic resources may be limited. Identifying cases before they evolve into complicated appendicitis, defined by necrosis, perforation, abscess formation or peritonitis [5], is critical, as these complications are associated with increased morbidity, prolonged hospitalization and higher healthcare costs [6]. The early recognition and appropriate management of complicated appendicitis significantly improve outcomes by minimizing these adverse effects [6].

Several scoring systems have been developed to aid in the diagnosis of appendicitis, such as the Alvarado score, the Pediatric Appendicitis Score (PAS) and the Appendicitis Inflammatory Response (AIR) score [7,8,9]. While these tools are useful for stratifying the likelihood of appendicitis in children, their reliability in distinguishing between uncomplicated and complicated cases is limited [10,11]. Factors such as interobserver variability, reliance on subjective parameters and inconsistent sensitivity in pediatric populations all contribute to their limitations. Thus, the development of alternative or adjunctive diagnostic markers is essential for improving risk stratification.

While imaging modalities like ultrasound (US) and computed tomography (CT) are frequently employed to confirm a diagnosis of appendicitis, their utility in children has limitations. US, though non-invasive and radiation-free, is operator-dependent and may yield inconclusive results, particularly in cases with atypical presentation [12]. CT scans offer higher diagnostic accuracy; however, concerns about ionizing radiation exposure and the associated long-term cancer risk make CT less desirable in the pediatric population [13]. These challenges underscore the need for effective, non-invasive and readily available diagnostic tools.

Hyponatremia, defined as a serum sodium concentration of less than 135 mmol/L [14], has emerged as a potential inflammatory marker in various conditions. The pathophysiologic process of the development of hyponatremia in inflammation is thought to be caused by the non-osmotic stimulation of the supraoptic and paraventricular nuclei of the hypothalamus by inflammatory cytokines, which causes the release of antidiuretic hormone (ADH). In turn, ADH leads to the insertion of aquaporins to the distal convoluted tubules and the collecting ducts of nephrons, causing dilutional hyponatremia [15].

The usefulness of this easily obtainable laboratory test in determining complicated cases of AA, alone or in combination with other inflammatory markers, has already been examined in adult populations, with promising results [16]. The aim of this study was to examine this interesting and novel parameter in a pediatric setting.

## 2. Materials and Methods

### 2.1. Data Collection

A retrospective review of hospital records from the 1st Pediatric Surgery Unit of Panagiotis and Aglaia Kyriakou Children’s Hospital of Athens and the Pediatric Surgery Unit of the University Hospital of Alexandroupolis was conducted, collecting cases of acute appendicitis that proceeded to surgery between 2020 and 2024. The study was conducted in accordance with the STROBE (Strengthening the Reporting of Observational Studies in Epidemiology) guidelines [17]. Ethical approval was obtained from the ethics committees of Panagiotis and Aglaia Kyriakou Children’s Hospital of Athens and of the University Hospital of Alexandroupolis (Protocol Numbers: 27/21-01-2025, 18924/08-04-2024, respectively). Patient records were retrospectively reviewed, and data were anonymized for analysis. For patients enrolled after the start of the research project, informed consent was obtained, as part of the procedures were linked to a doctoral thesis research protocol.

Surgical records were reviewed to identify appendectomies performed in the two centers between 2020 and 2024. A total of 664 surgeries were identified. From the above total, 9 cases were excluded as incidental appendectomies performed during surgical interventions for other pathologies and 164 were excluded because of a lack of laboratory data, as they were handled before the complete digitalization of patient records. For each of the included patients, preoperative blood work results were obtained, which included white blood cell (WBC) count, C-reactive protein (CRP), sodium (Na), potassium (K), urea, creatinine and platelet count.

Surgical and pathology reports of the identified cases were examined in order to assess the intraoperative findings. These were used to classify each case as uncomplicated or complicated. Complicated appendicitis was defined as the presence of necrosis, perforation, abscess formation or generalized peritonitis, in accordance with standard definitions in the literature [5]. Uncomplicated appendicitis included inflamed, non-perforated appendices without signs of necrosis or peritonitis [5].

Hyponatremia was defined as a serum sodium concentration below 135 mmol/L, following commonly accepted clinical cutoffs [14].

The majority of patients included were treated using the open surgical technique, which is the standard procedure in both participating centers, while a limited number of patients were treated laparoscopically. Intraoperative findings, as is mentioned above, were used to classify cases as uncomplicated or complicated, but all laboratory findings examined were obtained preoperatively. Therefore, the choice of operative technique was not considered to influence the primary outcomes of the present study and was excluded from our analysis. Furthermore, postoperative sodium levels were not assessed, as this was a retrospective study relying on available preoperative records. In many cases, particularly among patients with an uneventful recovery, postoperative laboratory tests are not performed, and patients are discharged within the first 24 to 48 h. As a result, we were unable to evaluate whether the normalization of sodium levels correlated with the resolution of inflammation in the postoperative period.

### 2.2. Statistical Analysis

The quantitative variables used in this study were presented as the median (interquartile range). Qualitative variables were expressed as the frequency (N) and percentage (%). The normal distribution of the data were assessed using the Kolmogorov–Smirnov test. Patient characteristics were compared using the non-parametric Mann–Whitney U test. Pearson’s Chi-square test or Fisher’s exact test were used to analyze qualitative variables. A receiver operating characteristic (ROC) curve was used to determine diagnostic accuracy of laboratory parameters (area under the curve (AUC)). The maximum point of the Youden index was adopted as the optimal cutoff point. Univariate and multivariate logistic regression analyses were conducted to explore the laboratory measurements associated with the histopathological diagnosis of acute complicated appendicitis. Odds ratios were calculated with 95% confidence intervals. Statistical analyses were performed using IBM SPSS Statistics 25.0. Values of *p* ≤ 0.05 were considered statistically significant.

## 3. Results

A total of 491 patient records were ultimately included and the available data are summarized in Table 1 and Table 2. A total of 25.5% of the patients were diagnosed with ACA and 74.5% with AUA. The results showed that the majority of the sample were males (60.3%). Patients with complicated appendicitis were younger (*p* = 0.01). Patients with complicated appendicitis had significantly higher levels of C-reactive protein (CRP) (4.7 vs. 1 mg/dL, *p* < 0.001), platelet counts (PLTs) (317 × 10^9^ vs. 286 × 10^9^ PLTs per microliter of blood, *p* = 0.001), white blood cell counts (WBCs) (17.2 × 10^3^ vs. 14 × 10^3^ WBCs per microliter of blood, *p* < 0.001) and neutrophil percentages (NEUT) (82.7% vs. 77%, *p* < 0.001). In contrast, ACA patients showed significantly lower creatinine (0.58 vs. 0.60 mg/dL, *p* = 0.005), sodium (Na) (136 vs. 138 mmol/L, *p* < 0.001), hemoglobin (Hb) (12.8 vs. 13.1 g/dL, *p* = 0.022) and hematocrit (Hct) (38% vs. 38.5%, *p* = 0.022) levels and lymphocyte percentages (LYM) (9.5% vs. 15.6%, *p* < 0.001) compared to AUA patients. A significantly higher percentage of ACA patients had hyponatremia, with Na lower than 135 mmol/L (38.4% vs. 2.2%, *p* < 0.001). No statistically significant differences were found in terms of gender (*p* = 0.160), urea (*p* = 0.055), K (*p* = 0.149) and MPV (*p* = 0.057) between the two groups.

**Figure 1:**
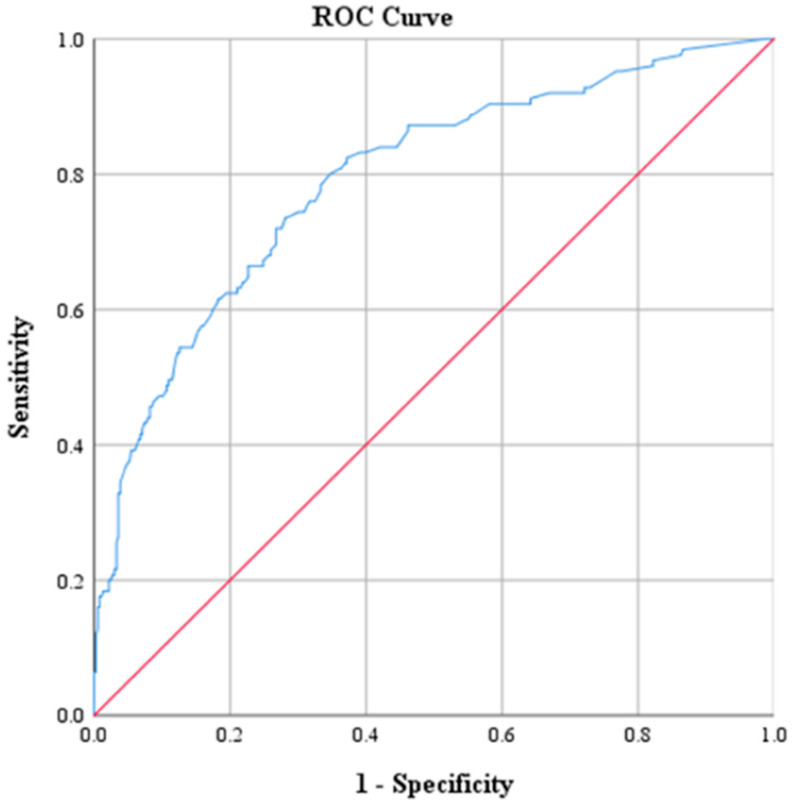
Receiver operating characteristic curve (ROC) of CRP.

**Figure 2:**
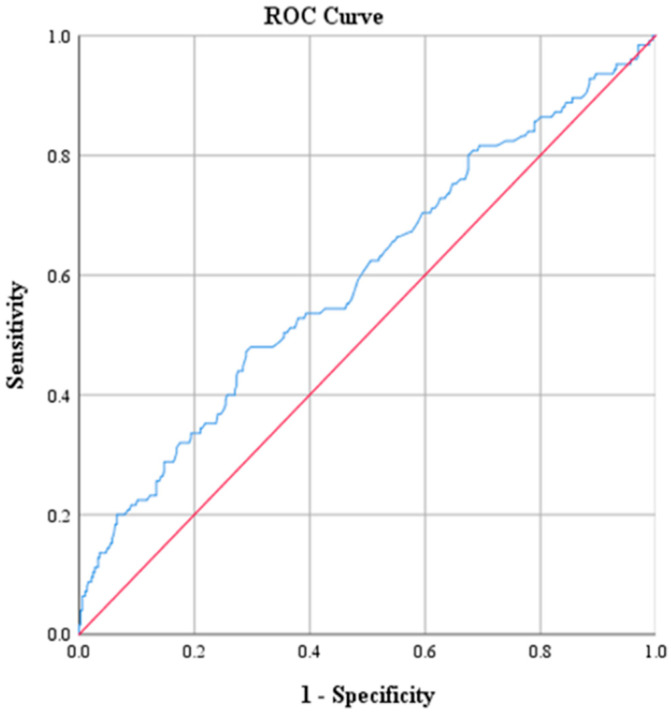
Receiver operating characteristic curve (ROC) of PLT.

**Figure 3:**
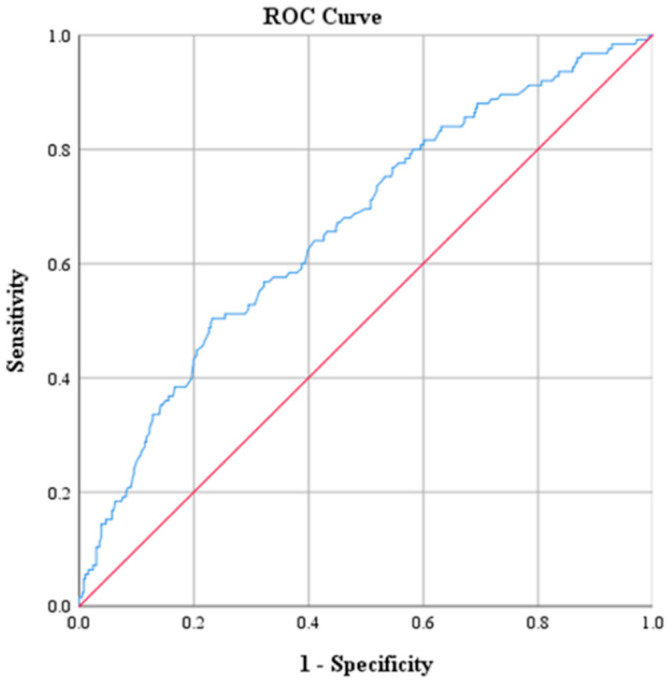
Receiver operating characteristic curve (ROC) of WBC.

**Figure 4:**
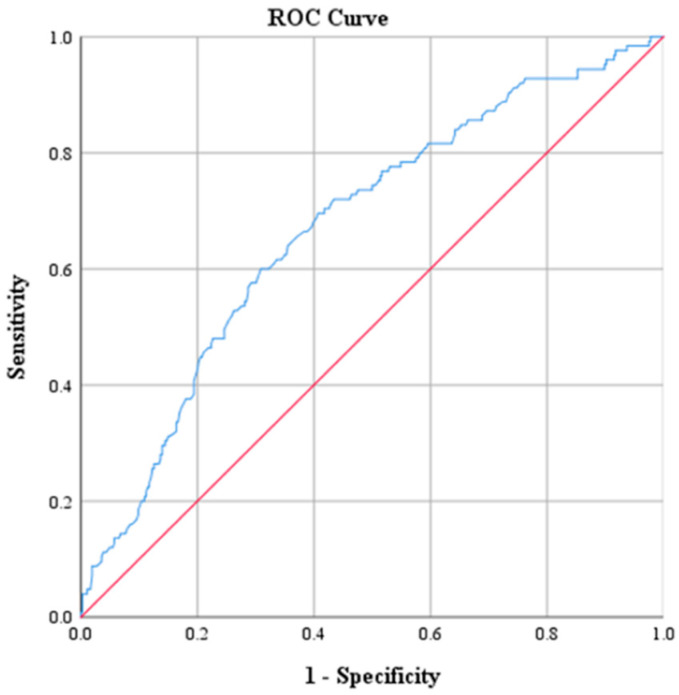
Receiver operating characteristic curve (ROC) of NEUT.

**Figure 5:**
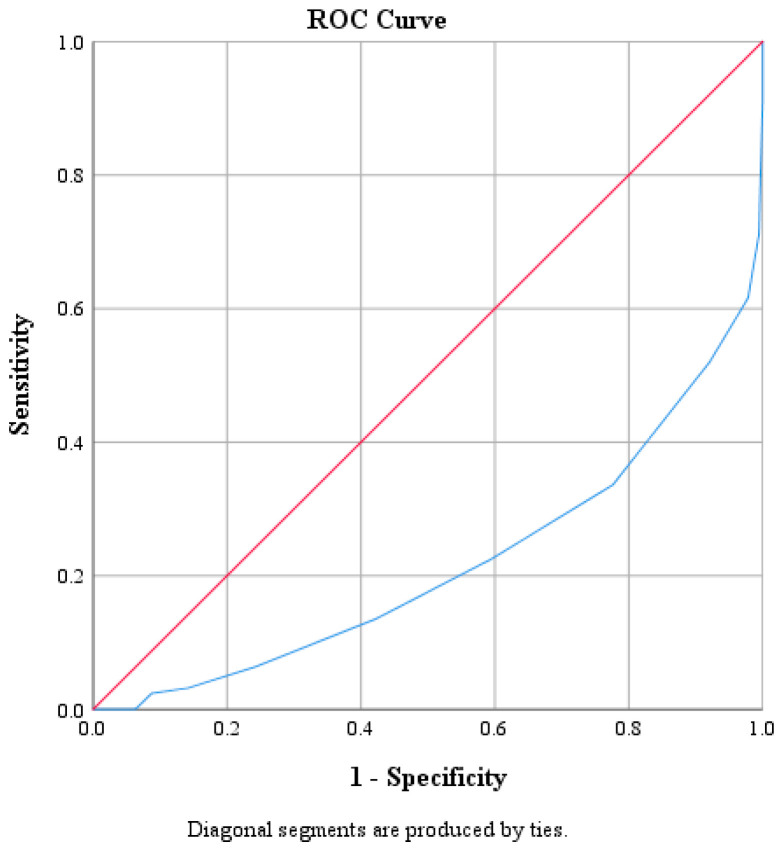
Receiver operating characteristic curve (ROC) of Na.

ROC curve analysis was performed to determine optimal cutoff points for CRP, Na, PLT, WBC and NEUT (Table 3 and Table 4). The optimal threshold of CRP for identifying patients with ACA was found to be above 2.17. This cutoff value was determined by the best combination of sensitivity and specificity, which were 73.6% and 71.9%, respectively. The corresponding positive predictive value (PPV) was 47.2%, and the negative predictive value (NPV) was approximately 88.9%. The AUC derived from the ROC was 0.793 with 95% CI: 0.746–0.840 (Figure 1). The optimal threshold of PLT was 330.5 × 10^9^ PLTs per microliter of blood, with a sensitivity of 47.2% and specificity of 71%. The corresponding PPV was 35.8% and the NPV was approximately 79.8%. The AUC derived from the ROC was 0.595 with 95% CI: 0.536–0.655 (Figure 2). Moreover, the optimal threshold of WBC was found to be above 17.15 × 10^3^ WBCs per microliter of blood. This cutoff value achieved a sensitivity of 50.4% and specificity of 76.8%. The corresponding PPV was 42.6%, and the NPV was approximately 81.9%. The AUC derived from the ROC was 0.664 with 95% CI: 0.609–0.719 (Figure 3). The optimal threshold of NEUT was established to be above 81.05%. This cutoff value yielded a sensitivity of 60% and a specificity of 69.1%. The corresponding PPV was 39.9% and the NPV was approximately 83.5%. The AUC derived from the ROC was 0.671 with 95% CI: 0.617–0.726 (Figure 4). Finally, the optimal threshold of Na was established at less than 135 mmol/L. This cutoff value yielded a sensitivity of 48% and a specificity of 92.1%. The corresponding PPV was 85.7% and the NPV was approximately 82.3%. The AUC derived from the ROC was 0.784 with 95% CI: 0.733–0.834 (Figure 5).

Univariate and multivariate logistic models were performed to determine the significant predictors of acute complicated appendicitis. The results revealed that patients with CRP higher than 2.17 mg/dL had a significantly higher likelihood of experiencing ACA [OR = 5.71, CI = 3.35–9.73, *p* < 0.001]. Moreover, hyponatremia (<135) was associated with a higher risk of ACA [OR = 18.30, CI = 7.67–43.63, *p* < 0.001]. Finally, patients with NEUT higher than 81.05% had a significantly higher likelihood of experiencing ACA [OR = 3.08, CI = 1.72–5.53, *p* < 0.001] (Table 5).

## 4. Discussion

AA remains a common surgical emergency in the pediatric population, with a significant contribution to pediatric morbidity. The pathophysiology of appendicitis begins with the luminal obstruction of the appendix, most often due to a fecalith. Obstruction leads to increased intraluminal pressure, venous congestion and subsequent ischemia, which promotes bacterial overgrowth and transmural inflammation. The clinical course of this progression can range from a mild, self-limiting condition to severe, life-threatening cases involving rupture, peritonitis, sepsis and even death [3]. Due to the above-mentioned variability in the expected outcomes, an accurate diagnosis accompanied by effective risk stratification is crucial for determining optimal therapeutic decisions. However, due to the variability in clinical presentations of acute appendicitis, especially in children, and despite advancements in imaging techniques and laboratory investigations, accurately identifying cases of complicated appendicitis remains a challenge [18]. This necessitates a search for novel methods of diagnosing, but mainly of stratifying, cases of pediatric acute appendicitis in order to identify complicated cases in a timely manner and avoid the increased morbidity, prolonged hospital stay and increased healthcare costs that are typical of complicated cases [19].

Several clinical scoring systems have been developed to aid in the diagnosis of appendicitis, particularly in children. The Alvarado score, the Pediatric Appendicitis Score (PAS) and the Appendicitis Inflammatory Response (AIR) score are among the most commonly utilized [7,8,9]. These systems combine clinical, laboratory and occasionally imaging parameters to stratify patients into low, intermediate or high risk for appendicitis. For example, the PAS incorporates symptoms such as the migration of pain, anorexia and fever, as well as laboratory markers such as leukocytosis and neutrophilia. While these scoring systems demonstrate high sensitivity, particularly in ruling out appendicitis in low-risk cases, their specificity is sub-optimal [20]. Factors such as reliance on subjective clinical signs, interobserver variability and age-related differences in symptom presentation contribute to their inconsistency [21]. For example, younger children may lack the ability to articulate classic symptoms like pain migration, leading to lower scores despite significant pathology [22]. Moreover, these scoring systems may not adequately account for atypical presentations or conditions that mimic appendicitis, such as mesenteric adenitis or gastrointestinal infections [22]. This highlights the need for adjunctive diagnostic tools to enhance specificity and improve our ability to distinguish complicated cases. In this particular study, the analysis of laboratory markers from our two pediatric surgery centers identified hyponatremia as a more significant marker of complicated appendicitis compared to more commonly used laboratory parameters such as C-reactive protein (CRP) and neutrophil count. This lends weight to the hypothesis that the inclusion of hyponatremia in scoring systems incorporating CRP and neutrophil count, such as the AIR score, may both improve the diagnostic accuracy of the scoring system and modify it into a tool for the stratification of cases into complicated and uncomplicated.

The importance of the pivotal role of imaging studies in the evaluation of suspected appendicitis, particularly when clinical findings are equivocal, must not be understated. US is often the first-line imaging modality in children due to its safety profile, availability and lack of ionizing radiation. The American College of Radiology recommends US as the initial imaging study for pediatric patients with suspected appendicitis due to its non-invasive nature and absence of radiation exposure [23]. Key sonographic findings include a non-compressible, dilated appendix with a diameter >6 mm, periappendiceal fluid and the increased echogenicity of the surrounding fat [24]. However, US is highly operator-dependent, and its diagnostic accuracy is influenced both by the skill of the technician and by patient factors, such as body type and the presence of bowel gas [12]. Inconclusive US findings are not uncommon, necessitating further diagnostic evaluation. CT provides superior sensitivity and specificity compared to US and is considered the gold standard for diagnosing appendicitis in adults. Studies have reported CT sensitivities ranging from 88% to 100%, and specificities from 91% to 99% [25]. However, the routine use of CT in children is limited by concerns about radiation exposure, which carries a potential long-term risk of malignancy [13]. Efforts to reduce radiation doses through low-dose protocols have shown promise, but the use of CT remains a topic of debate, particularly for repeated imaging in recurrent or atypical cases [26]. Magnetic resonance imaging (MRI) offers a radiation-free alternative with comparable diagnostic accuracy, though its use is limited by cost, availability and longer imaging times; it may therefore be unsuitable, especially for younger patients [27]. These limitations underscore the importance of integrating imaging findings with clinical and laboratory data to optimize diagnostic accuracy in children. The fact that hyponatremia was a more significant marker of complicated appendicitis than established laboratory markers in our study leads us to believe that hyponatremia may serve as an adjunctive tool to improve risk stratification and guide decision making regarding the need for advanced imaging studies. For example, in cases where ultrasound findings are inconclusive, the presence of hyponatremia could support a higher suspicion for complicated appendicitis and justify the use of advanced imaging modalities.

Hyponatremia has already been implicated as a marker of disease severity in various conditions. In pneumonia, for instance, hyponatremia correlates with higher inflammatory marker concentrations and worse clinical outcomes, serving as an indicator of disease severity [28]. Similarly, in conditions like sepsis, heart failure and liver cirrhosis, hyponatremia reflects underlying inflammatory response processes and is an indicator of disease outcome [29,30,31]. In the perioperative setting, hyponatremia has been associated with increased morbidity and mortality. In a recent systematic review focusing on adult patients with preoperative hyponatremia, a 2-fold increase in early mortality and a 2.5-fold increase in the risk of major complications was found in patients with preoperative hyponatremia [32]. In the same adult study, hyponatremia was found to have an 88% specificity and 25% sensitivity for predicting major complications [32]. These findings are in line with those of the present study, in which we have shown an association of preoperative hyponatremia with an increase in the likelihood of complicated appendicitis.

**Figure 6:**
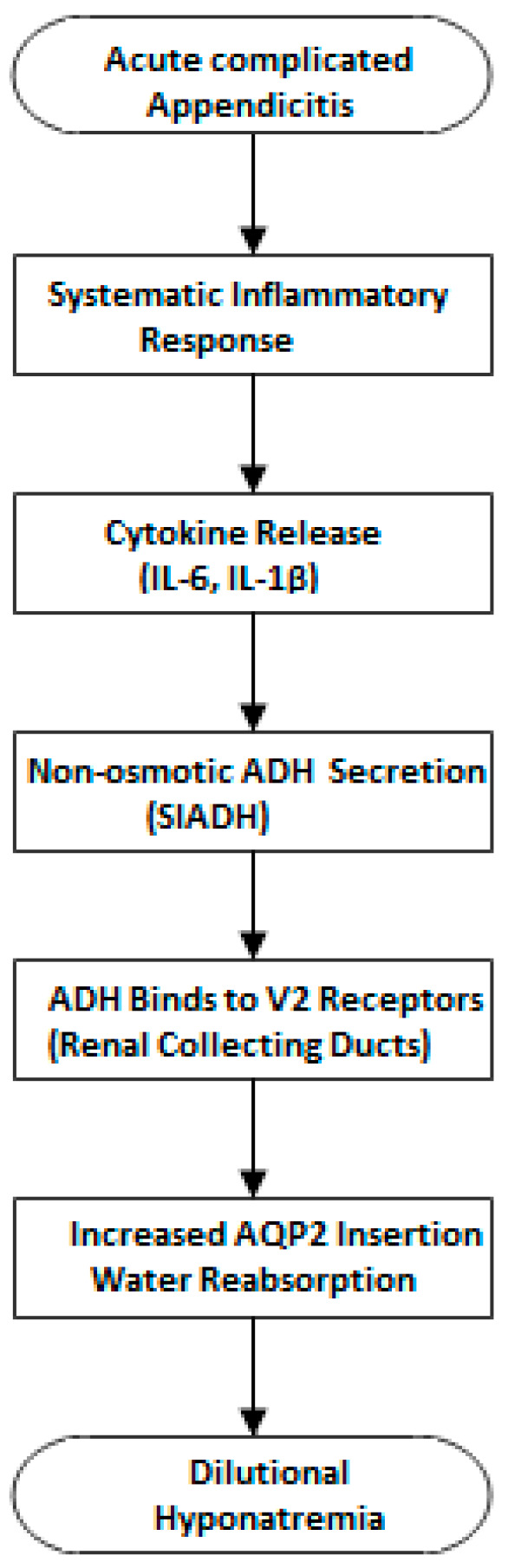
Suspected pathway of hyponatremia development in AA.

Despite its apparent role in inflammation, the exact pathophysiology underlying the development of hyponatremia in inflammatory conditions, as well as its impact on clinical outcomes, remains under discussion. A key proposed mechanism involves the syndrome of inappropriate antidiuretic hormone secretion (SIADH) (Figure 6), in which excessive ADH release leads to impaired free water excretion and dilutional hyponatremia, even in the absence of hypovolemia or osmotic triggers [15]. This occurs through ADH binding to vasopressin 2 (V2) receptors in the renal collecting ducts, triggering intracellular signaling cascades that result in the insertion of aquaporin-2 (AQP2) water channels into the apical membrane [33]. The increased water permeability facilitates excessive water reabsorption, diluting serum sodium concentrations while also leading to high urine sodium excretion due to continued sodium reabsorption in the proximal tubule and persistent natriuresis [15]. Inflammatory cytokines play a central role in this process, with interleukin-6 (IL-6) and interleukin-1β (IL-1β) implicated in stimulating hypothalamic ADH secretion. IL-6, in particular, has been shown to enhance ADH release independently of plasma osmolality, while IL-1β contributes to the systemic inflammatory response by disrupting normal renal sodium handling [12,34]. The combined effects of these cytokines in systemic inflammation can lead to a self-perpetuating cycle of water retention and sodium imbalance, contributing to the hyponatremia observed in various inflammatory and infectious diseases. However, it must be emphasized that while this mechanism is biologically plausible and supported by the literature, it remains hypothetical in the context of our study, as we did not directly measure ADH or cytokine levels.

Our study reinforces the emerging role of hyponatremia as a potential biomarker for complicated appendicitis. Patients with acute complicated appendicitis (ACA) exhibited significantly lower sodium levels compared to those with acute uncomplicated appendicitis (AUA), with a notable proportion presenting with sodium levels below 135 mmol/L. Although the median sodium difference between ACA and AUA was modest (136 vs. 138 mmol/L), and both values fall within the normal range (135–145 mmol/L), the difference was statistically significant. This suggests that even subtle reductions in sodium, while not alarming on their own, may carry diagnostic significance when interpreted alongside other inflammatory markers. For sodium values <135 mmol/L, the sensitivity and specificity were calculated at 48% and 92.1%, respectively, with a positive predictive value (PPV) of 85.7% and a negative predictive value (NPV) of 82.3%. This indicates that while hyponatremia alone may not detect all cases of ACA, its high specificity and PPV make it a particularly strong indicator when present. In other words, a child with a sodium level below 135 mmol/L is significantly more likely to have complicated appendicitis than not. The AUC of 0.784 further supports serum sodium as a moderately accurate tool for discriminating between ACA and AUA in children. Additionally, our analysis identified age as a significant predictor of ACA (*p* = 0.01). This is consistent with the existing literature [35], which shows that younger children are more likely to present with delayed or atypical symptoms due to limitations in communication and less specific clinical signs. These delays contribute to disease progression and the development of complications before diagnosis and management. This finding further underscores the importance of objective laboratory markers, such as serum sodium, in guiding early clinical decision making—especially in younger, less communicative children.

When hyponatremia is considered alongside other inflammatory markers, such as CRP and neutrophil count, the predictive value for ACA is enhanced further. In our study, CRP levels above 2.17 mg/dL and neutrophil percentages above 81% were also significantly associated with ACA, strengthening the argument that a combination of these biomarkers could improve risk stratification. Collectively, these findings highlight the potential of incorporating hyponatremia into existing clinical scoring systems for appendicitis severity in children. Given its strong association with complicated appendicitis, even modest reductions in serum sodium could serve as an early warning sign prompting more urgent surgical evaluation or imaging, particularly when combined with elevated levels of CRP and neutrophilia. While our study focused on the pediatric population, similar associations have been reported in adults. For instance, Kim et al. [16] demonstrated that hyponatremia was an independent predictor of perforated appendicitis in adults, suggesting that the prognostic value of serum sodium may extend across age groups. However, further validation is needed for integration in pediatric risk stratification algorithms. Our study has some limitations. First, as a retrospective study, it is inherently subject to biases related to data collection and missing information. It is also important to address the fact that 164 cases were excluded due to incomplete digital records. This data loss occurred during the transition to electronic medical records, and was not related to disease severity or clinical presentation. All patients with suspected appendicitis received preoperative blood testing as part of a standard operating protocol. Thus, missing data were due to documentation issues rather than case selection. Consequently, we believe the exclusion of these cases was random and unlikely to have introduced systematic selection bias.

Another limitation relates to the timing of sodium measurement. Serum sodium levels were obtained from the initial preoperative blood work, typically at the time of admission. However, as this is a retrospective study, the precise timing in relation to symptom onset or disease progression could not be assessed. This may have introduced variability, as prolonged inflammation prior to blood sampling could contribute to more pronounced hyponatremia. Despite this, our approach reflects routine clinical practice. Future prospective studies could better standardize the timing of laboratory measurements to more accurately characterize the association between inflammation and electrolyte disturbances.

Additionally, this is not the first study to investigate hyponatremia as a marker in pediatric acute complicated appendicitis. However, our study adds to the existing literature by analyzing a large cohort of patients from two major pediatric surgery centers in Greece, strengthening the reliability of our findings.

## 5. Conclusions

Our study reinforces the association between hyponatremia and acute complicated appendicitis in pediatric patients. Among the various laboratory markers analyzed, preoperative hyponatremia was significantly associated with disease severity, with lower sodium levels observed in patients with complicated appendicitis. Although the median sodium difference was modest, hyponatremia demonstrated a high specificity and positive predictive value. When combined with other inflammatory markers, such as CRP and neutrophil count, hyponatremia further enhanced risk stratification, potentially aiding in the earlier identification of high-risk cases. Given the importance of the timely surgical management of such cases, incorporating serum sodium levels into the preoperative assessment of pediatric appendicitis may serve as a valuable adjunct in clinical decision making. Further studies are needed to validate these findings and clarify the clinical utility of serum sodium in pediatric risk assessment algorithms, as well as to further clarify the pathophysiological mechanisms linking inflammation and the development of hyponatremia.

## Figures and Tables

**Table 1:** Distribution of sex and age in total sample and by severity classification.

Characteristic	Total (*N* = 491)	AUA (*N* = 366)	ACA (*N* = 125)	*p*
Sex (*N*, %)				0.160
Male	296 (60.3)	214 (58.5)	82 (65.6)	
Female	195 (39.7)	152 (41.5)	43 (34.4)	
Age (years)	10.78 (3.05)	11.02 (2.86)	10 (3.46)	0.010
Min–Max	1.5–16	4–16	1.5–15	

Mean (SD).

**Table 2:** Laboratory results in total sample and by severity classification.

Characteristic	Total (*N* = 491)	AUA (*N* = 366)	ACA (*N* = 125)	*p*
CRP (mg/dL)	1.5 (0.5–4)	1 (0.4–2.61)	4.7 (2–9.17)	<0.001
Urea (mmol/L)	23 (19–27.25)	24 (20–28)	22 (19–27)	0.055
Creatinine (mg/dL)	0.6 (0.5–0.7)	0.6 (0.5–0.7)	0.58 (0.43–0.7)	0.005
K (mmol/L)	4.3 (4–4.5)	4.3 (4–4.6)	4.2 (3.93–4.5)	0.149
Na (mmol/L)	137 (136–139)	138 (137–139)	136 (133–137)	<0.001
Hyponatremia (<135)				<0.001
Yes	56 (11.4%)	8 (2.2%)	48 (38.4%)	
No	435 (88.6%)	358 (97.8%)	77 (61.6%)	
Hb (g/dL)	13.1 (12.4–13.8)	13.1 (12.5–13.9)	12.8 (12–13.75)	0.022
Ht	38.4 (36.6–40.6)	38.5 (36.9–40.6)	38 (35.9–40.05)	0.022
PLT (×10^9^ PLTs per microliter of blood)	291 (243–355)	286 (242–347)	317 (258–389)	0.001
MPV (fl)	10 (9.4–10.6)	10 (9.5–10.6)	9.9 (9.2–10.5)	0.057
WBC (×10^3^ WBCs per microliter of blood)	14.5 (10.8–17.82)	14 (10.22–17)	17.2 (13.4–19.8)	<0.001
NEUT	78.5 (69.6–84.5)	77 (67.5–82.7)	82.7 (76.7–87.7)	<0.001
LYM	14.1 (8.8–21.3)	15.6 (10.38–23.03)	9.5 (6.2–14.8)	<0.001

Median (interquartile range; 25th–75th percentile).

**Table 3:** Area under the curve values.

	AUC Value	*p*	95% CI
CRP	0.793	<0.001	0.746, 0.840
Urea	0.442	0.055	0.383, 0.502
Creatinine	0.417	0.006	0.357, 0.476
K	0.457	0.150	0.397, 0.516
Na	0.784	<0.001	0.733, 0.834
Hb	0.432	0.023	0.370, 0.493
Ht	0.431	0.022	0.370, 0.493
PLT	0.595	0.001	0.536, 0.655
MPV	0.443	0.057	0.382, 0.504
WBC	0.664	<0.001	0.609, 0.719
NEUT	0.671	<0.001	0.617, 0.726
LYM	0.297	<0.001	0.244, 0.349

**Table 4:** Cutoff values for significant parameters in prediction of acute complicated appendicitis.

Variable	Cutoff Points	Sensitivity	Specificity	PPV	NPV	AUC
CRP (mg/dL)	2.17	73.6	71.9	47.2	88.9	0.793
PLT	330.5	47.2	71.0	35.8	79.8	0.595
WBC	17.15	50.4	76.8	42.6	81.9	0.664
NEUT	81.05	60.0	69.1	39.9	83.5	0.671
Na (mmol/L)	135	48.0	92.1	85.7	82.3	0.784

**Table 5:** Multivariate logistic regression model to predict the risk of acute complicated appendicitis.

	Univariate Logistic Regression		Multivariate Logistic Regression	
	OR (95% CI)	*p*	OR (95% CI)	*p*
CRP ≥ 2.175	7.12 (4.5–11.26)	<0.001	5.71 (3.35–9.73)	<0.001
Hyponatremia (<135), Yes	27.9 (12.69–61.34)	<0.001	18.30 (7.67–43.63)	<0.001
PLT ≥ 330.5	2.19 (1.44–3.33)	<0.001	1.43 (0.83–2.46)	0.195
WBC ≥ 17.15	3.36 (2.19–5.15)	<0.001	1.76 (0.96–3.23)	0.066
NEUT ≥ 81.05	3.36 (2.2–5.12)	<0.001	3.08 (1.72–5.53)	<0.001

Note: Analyses are adjusted for sex and age, OR = odds ratio, CI = confidence interval, *p* < 0.05.

## Data Availability

The database of the study can be accessed upon request at the address kampouri@med.duth.gr.

## References

[1] Buckius M.T., McGrath B., Monk J., Grim R., Bell T., Ahuja V. (2012). Changing epidemiology of acute appendicitis in the United States: Study period 1993–2008. J. Surg. Res..

[2] Jumah S., Wester T. (2022). Non-operative management of acute appendicitis in children. Pediatr. Surg. Int..

[3] Lotfollahzadeh S., Lopez R.A., Deppen J.G. (2024). Appendicitis. StatPearls.

[4] Risteski T., Sokolova R., Memeti S., Simeonov R. (2022). Laparoscopic versus open appendectomy in pediatric patients: Operative and postoperative experience. J. Clin. Trials Exp. Investig..

[5] Mariage M., Sabbagh C., Grelpois G., Prevot F., Darmon I., Regimbeau J.-M. (2019). Surgeon’s Definition of Complicated Appendicitis: A Prospective Video Survey Study. Euroasian J. Hepato-Gastroenterol..

[6] Kong V., Aldous C., Handley J., Clarke D. (2013). The cost effectiveness of early management of acute appendicitis underlies the importance of curative surgical services to a primary healthcare programme. Ann. R. Coll. Surg. Engl..

[7] El Hattabi K., Bouali M., El Berni Y., Bensardi F., El Bakouri A., Moufakkir A., Riad N., Fadil A. (2022). Value of Alvarado scoring system in diagnosis of acute appendicitis. Ann. Med. Surg..

[8] Parveen K.Z., Avabratha K.S., Shetty K. (2017). Pediatric appendicitis score in the diagnosis of childhood appendicitis: A validation study. Int. J. Contemp. Pediatr..

[9] Andersson M., Kolodziej B., Andersson R.E. (2021). Validation of the Appendicitis Inflammatory Response (AIR) Score. World J. Surg..

[10] Haak F., Kollmar O., Ioannidis A., Slotta J.E., Ghadimi M.B., Glass T., von Strauss Und Torney M. (2022). Predicting complicated appendicitis based on clinical findings: The role of Alvarado and Appendicitis Inflammatory Response scores. Langenbeck’s Arch. Surg..

[11] Edgar S.N. (2024). Effectiveness of the PAS Scale for Diagnosing the Severity of Acute Appendicitis in Children: A Cohort Study. medRxiv.

[12] Trout A.T., Sanchez R., Ladino-Torres M.F., Pai D.R., Strouse P.J. (2012). A critical evaluation of US for the diagnosis of pediatric acute appendicitis in a real-life setting: How can we improve the diagnostic value of sonography?. Pediatr. Radiol..

[13] Harris E. (2023). Radiation From CT Scans in Young People Tied to Higher Cancer Risk. JAMA.

[14] Spasovski G., Vanholder R., Allolio B., Annane D., Ball S., Bichet D., Decaux G., Fenske W., Hoorn E.J., Ichai C. (2014). Clinical practice guideline on diagnosis and treatment of hyponatraemia. Nephrol. Dial. Transplant..

[15] Swart R.M., Hoorn E.J., Betjes M.G., Zietse R. (2010). Hyponatremia and Inflammation: The Emerging Role of Interleukin-6 in Osmoregulation. Nephron Physiol..

[16] Kim D.Y., Nassiri N., de Virgilio C., Ferebee M.P., Kaji A.H., Hamilton C.E., Saltzman D.J. (2015). Association Between Hyponatremia and Complicated Appendicitis. JAMA Surg..

[17] Cuschieri S. (2019). The Strobe guidelines. Saudi J. Anaesth..

[18] Lounis Y., Hugo J., Demarche M., Seghaye M.-C. (2020). Influence of age on clinical presentation, diagnosis delay and outcome in pre-school children with acute appendicitis. BMC Pediatr..

[19] Baxter K.J., Nguyen H.T., Wulkan M.L., Raval M.V. (2018). Association of Health Care Utilization with Rates of Perforated Appendicitis in Children 18 Years or Younger. JAMA Surg..

[20] Macco S., Vrouenraets B.C., de Castro S.M. (2016). Evaluation of scoring systems in predicting acute appendicitis in children. Surgery.

[21] Kulik D.M., Uleryk E.M., Maguire J.L. (2013). Does this child have appendicitis? A systematic review of clinical prediction rules for children with acute abdominal pain. J. Clin. Epidemiol..

[22] Almaramhy H.H. (2017). Acute appendicitis in young children less than 5 years: Review article. Ital. J. Pediatr..

[23] Crocker C., Akl M., Abdolell M., Kamali M., Costa A.F. (2020). Ultrasound and CT in the Diagnosis of Appendicitis: Accuracy with Consideration of Indeterminate Examinations According to STARD Guidelines. Am. J. Roentgenol..

[24] Jacob D.K. (2025). Acute appendicitis | Radiology Reference Article. Radiopaedia.org. https://radiopaedia.org/articles/acute-appendicitis.

[25] Leite N.P., Pereira J.M., Cunha R., Pinto P., Sirlin C. (2005). CT Evaluation of Appendicitis and Its Complications: Imaging Techniques and Key Diagnostic Findings. Am. J. Roentgenol..

[26] Zacharias C., Alessio A.M., Otto R.K., Iyer R.S., Philips G.S., Swanson J.O., Thapa M.M. (2013). Pediatric CT: Strategies to Lower Radiation Dose. Am. J. Roentgenol..

[27] Wisnowski J.L., Sperling V.R., Panigrahy A., Blüml S., Panigrahy A. (2013). Challenges in Pediatric Magnetic Resonance Imaging. MR Spectroscopy of Pediatric Brain Disorders.

[28] Królicka A.L., Kruczkowska A., Krajewska M., Kusztal M.A. (2020). Hyponatremia in Infectious Diseases—A Literature Review. Int. J. Environ. Res. Public Health.

[29] Kumar S., Pratima K., Bhattacharya R., Ambedkar S.N., Saini R.P. (2023). Hyponatremia in sepsis and its association with SOFA score: An observational cross-sectional study. J. Med. Sci. Res..

[30] Rodriguez M., Hernandez M., Cheungpasitporn W., Kashani K.B., Riaz I., Rangaswami J., Herzog E., Guglin M., Krittanawong C. (2019). Hyponatremia in Heart Failure: Pathogenesis and Management. Curr. Cardiol. Rev..

[31] John S., Thuluvath P.J. (2015). Hyponatremia in cirrhosis: Pathophysiology and management. World J. Gastroenterol..

[32] Teo C.B., Gan M.Y., Tay R.Y.K., Loh W.J., Loh N.-H.W. (2023). Association of Preoperative Hyponatremia with Surgical Outcomes: A Systematic Review and Meta-analysis of 32 Observational Studies. J. Clin. Endocrinol. Metab..

[33] Nielsen S., Frøkiær J., Marples D., Kwon T.-H., Agre P., Knepper M.A. (2002). Aquaporins in the Kidney: From Molecules to Medicine. Physiol. Rev..

[34] Landgraf R., Neumann I., Holsboer F., Pittman Q.J. (1995). Interleukin-1β Stimulates both Central and Peripheral Release of Vasopressin and Oxytocin in the Rat. Eur. J. Neurosci..

[35] Khan N., Khan M.A., Khan J., Ali S., Khattak I., Masood A. (2020). Frequency and outcome of complicated appendicitis in toddlers and preschoolers. J. Pediatr. Adolesc. Surg..

